# Bis[2-meth­oxy-6-(phenyl­iminiomethyl)phenolate-κ^2^ *O*,*O*′]bis­(thio­cyanato-κ*N*)manganese(II)

**DOI:** 10.1107/S1600536811009330

**Published:** 2011-03-19

**Authors:** Jin-Bei Shen, Guo-Di Ge, Guo-Liang Zhao

**Affiliations:** a College of Chemistry and Life Science, Zhejiang Normal University, Jinhua, Zhejiang 321004, People’s Republic of China; b Xingzhi College, Zhejiang Normal University, Jinhua, Zhejiang 321004, People’s Republic of China

## Abstract

The Mn^II^ atom in the title complex, [Mn(NCS)_2_(C_14_H_13_NO_2_)_2_], lies on a center of inversion in a MnO_4_N_2_ octa­hedral geometry. The Schiff base is present in its zwitterionic form and is *O*,*O*′-chelated to the metal atom. The imino N atom is protonated and is involved in an intra­molecular hydrogen bond with the phenolate O atom.

## Related literature

For Schiff base ligands derived from *o*-vanillin and aniline and their rare earth complexes, see: Li *et al.* (2008 ▶); Liu *et al.* (2009 ▶); Xian *et al.* (2008 ▶); Zhao *et al.* (2005 ▶, 2007 ▶).
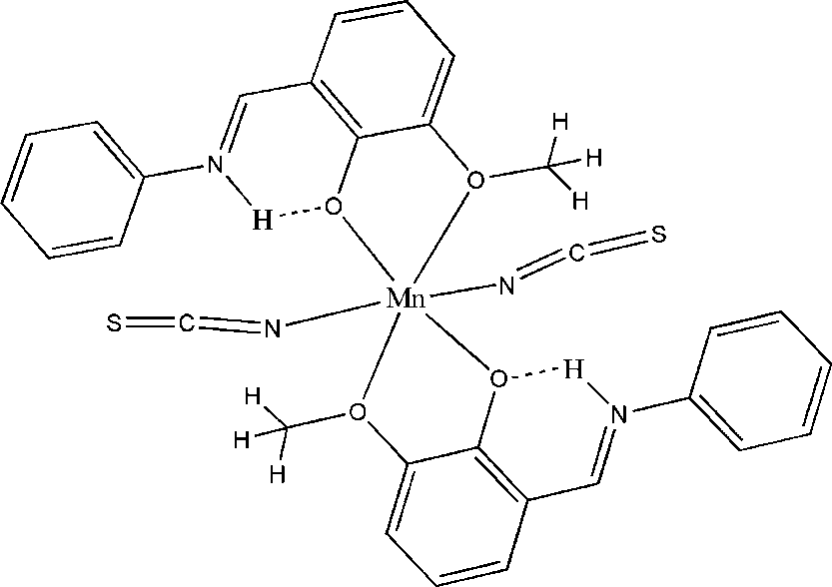


## Experimental

### 

#### Crystal data

[Mn(NCS)_2_(C_14_H_13_NO_2_)_2_]
*M* *_r_* = 625.61
Triclinic, 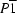

*a* = 9.0204 (2) Å
*b* = 9.3070 (2) Å
*c* = 9.4087 (2) Å
α = 87.417 (1)°
β = 82.010 (1)°
γ = 65.693 (1)°
*V* = 712.81 (3) Å^3^
*Z* = 1
Mo *K*α radiation
μ = 0.65 mm^−1^
*T* = 296 K
0.29 × 0.17 × 0.05 mm

#### Data collection

Bruker APEXII area-detector diffractometer
Absorption correction: multi-scan (*SADABS*; Sheldrick, 1996 ▶) *T*_min_ = 0.877, *T*_max_ = 0.970
9483 measured reflections
2509 independent reflections
2233 reflections with *I* > 2σ(*I*)
*R*_int_ = 0.022

#### Refinement

*R*[*F* ^2^ > 2σ(*F* ^2^)] = 0.028
*wR*(*F* ^2^) = 0.077
*S* = 1.06
2509 reflections
188 parameters
2 restraints
H-atom parameters constrained
Δρ_max_ = 0.17 e Å^−3^
Δρ_min_ = −0.24 e Å^−3^

### 

Data collection: *APEX2* (Bruker, 2006 ▶); cell refinement: *SAINT* (Bruker, 2006 ▶); data reduction: *SAINT*; program(s) used to solve structure: *SHELXS97* (Sheldrick, 2008 ▶); program(s) used to refine structure: *SHELXL97* (Sheldrick, 2008 ▶); molecular graphics: *SHELXTL* (Sheldrick, 2008 ▶); software used to prepare material for publication: *SHELXL97*.

## Supplementary Material

Crystal structure: contains datablocks I, global. DOI: 10.1107/S1600536811009330/ng5130sup1.cif

Structure factors: contains datablocks I. DOI: 10.1107/S1600536811009330/ng5130Isup2.hkl

Additional supplementary materials:  crystallographic information; 3D view; checkCIF report

## Figures and Tables

**Table 1:** Hydrogen-bond geometry (Å, °)

*D*—H⋯*A*	*D*—H	H⋯*A*	*D*⋯*A*	*D*—H⋯*A*
N1—H1*A*⋯O1	0.86	1.97	2.6501 (16)	135

**Fig. 1.**
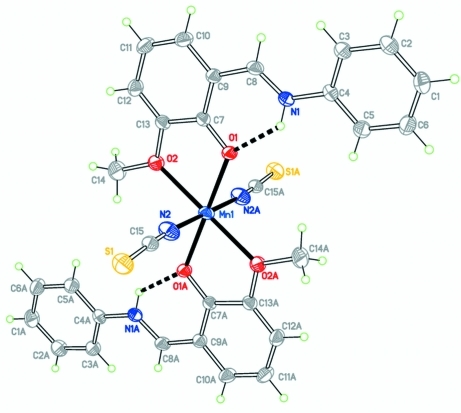
The molecular structure of the title complex, showing the atom-labelling scheme. Displacement ellipsoids are drawn at the 30% probability level.

**Fig. 2.**
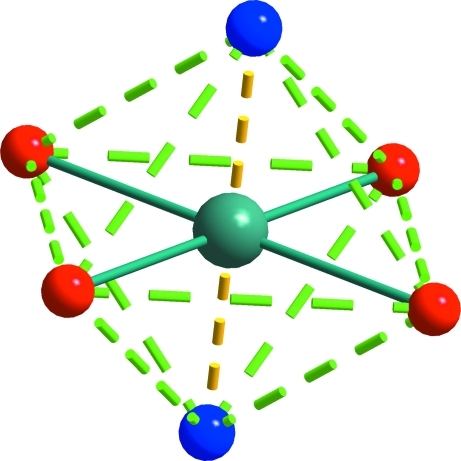
The coordination environment of the Mn(II).

**Fig. 3.**
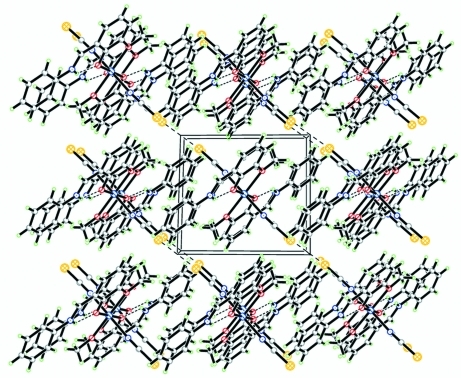
The stacking plot of the title compound, showing H-bond interactions (dashed lines) and π–π stacking interactions.

## supplementary crystallographic information

### Comment

For many years, there has been considerable interest in the study of Schiff base compounds due to their biological activity (Zhao *et al.*, 2005). Interested in this field, we have been synthesized several analogous Schiff bases derived from *o*-vanillin and prepared their transitional and rare metal complexes further. In a few of articles we have reported our partial research results (Zhao *et al.*, 2007; Xian *et al.* 2008; Li *et al.* 2008; Liu *et al.* 2009). Herein, we describe a new Mn(II) complex.

The structure of complex (1) was shown in Fig. 1 and the coordination environment of Mn(II) was shown in Fig. 2. In this complex the Mn(II) is six- coordinated by two N atoms from thiocyanate ions and four O atoms from the Schiff bases (HL), which can be described as a distorted octahedral geometry. There thiocyanate anions coordinate to Mn(II) ion with N atoms occupying the apices and two HL ligands chelate the Mn(II) ion with four O atoms from deprotonated phenol groups and methoxyl groups occupying the equatorial positions. The Mn—O and Mn—N bond distances were listed in Table 1, The distances between Mn(II) and methoxyl O atoms are obvious longer than Mn—O(phenolic) bond distances, which are similar to the analogous complexes (Zhao *et al.*, 2007; Li *et al.*, 2008, Liu *et al.*, 2009).

The hydrogen bonds and π–π weak non-covalent interactions lend stability to the structure. The stacking plot of this compound was shown in Fig. 3. In HL ligand, two protons of phenolic hydroxyl groups considered to have transferred to imine N atoms involve in forming intramolecular hydrogen bonds. The π–π interactions exist both intra and extra molecules between the approximate paralleled participating benzene rings, which may be the primary forces keep the complex molecules packing together.

### Experimental

Reagents and solvents used were of commercially available quality and without purified before using. The Schiff base ligand 2-(phenyliminomethyl)-6-methoxyphenol was synthesized from condensation of *o*-vanillin and aniline. The title compound was synthesized by traditional method. 1 mmol HL ligand was dissolved in ethanol, then 0.5 mmol Mn(NO_3_)_2_.6H_2_O (in ethanol) was added to the upper solution. The mixture solution was stirred for 2 h at room temperature. Furthermore, 1 mmol NH_4_SCN (dissolved in ethanol) was added. The mixture was stirred again for 8 h at room temperature. At last, deposit was filtered out and the reddish-brown solution was kept in the open air. The red crystal was obtained after several days.

### Refinement

The structure was solved by direct methods and successive Fourier difference synthesis. The H atoms bonded to C and N atoms were positioned geometrically and refined using a riding model [aliphatic C—H = 0.96 Å (*U*_iso_(H) = 1.5*U*_eq_(C)), aromatic C—H = 0.93 Å (*U*_iso_(H) = 1.2*U*_eq_(C)) and N—H = 0.86 Å with *U*_iso_(H) = 1.2*U*_eq_(N).

### Crystal data

**Table:**

[Mn(NCS)_2_(C_14_H_13_NO_2_)_2_]	*Z* = 1
*M**_r_* = 625.61	*F*(000) = 323
Triclinic, *P*1	*D*_x_ = 1.457 Mg m^−^^3^
Hall symbol: -P 1	Mo *K*α radiation, λ = 0.71073 Å
*a* = 9.0204 (2) Å	Cell parameters from 4271 reflections
*b* = 9.3070 (2) Å	θ = 2.2–25.0°
*c* = 9.4087 (2) Å	µ = 0.65 mm^−^^1^
α = 87.417 (1)°	*T* = 296 K
β = 82.010 (1)°	Block, red
γ = 65.693 (1)°	0.29 × 0.17 × 0.05 mm
*V* = 712.81 (3) Å^3^	

### Data collection

**Table:**

Bruker APEXII area-detector diffractometer	2509 independent reflections
Radiation source: fine-focus sealed tube	2233 reflections with *I* > 2σ(*I*)
graphite	*R*_int_ = 0.022
φ and ω scans	θ_max_ = 25.0°, θ_min_ = 2.2°
Absorption correction: multi-scan (*SADABS*; Sheldrick, 1996)	*h* = −10→10
*T*_min_ = 0.877, *T*_max_ = 0.970	*k* = −11→11
9483 measured reflections	*l* = −11→11

### Refinement

**Table:**

Refinement on *F*^2^	Primary atom site location: structure-invariant direct methods
Least-squares matrix: full	Secondary atom site location: difference Fourier map
*R*[*F*^2^ > 2σ(*F*^2^)] = 0.028	Hydrogen site location: inferred from neighbouring sites
*wR*(*F*^2^) = 0.077	H-atom parameters constrained
*S* = 1.06	*w* = 1/[σ^2^(*F*_o_^2^) + (0.0423*P*)^2^ + 0.1242*P*] where *P* = (*F*_o_^2^ + 2*F*_c_^2^)/3
2509 reflections	(Δ/σ)_max_ = 0.001
188 parameters	Δρ_max_ = 0.17 e Å^−^^3^
2 restraints	Δρ_min_ = −0.24 e Å^−^^3^

### Special details

**Table:**

Geometry. All e.s.d.'s (except the e.s.d. in the dihedral angle between two l.s. planes) are estimated using the full covariance matrix. The cell e.s.d.'s are taken into account individually in the estimation of e.s.d.'s in distances, angles and torsion angles; correlations between e.s.d.'s in cell parameters are only used when they are defined by crystal symmetry. An approximate (isotropic) treatment of cell e.s.d.'s is used for estimating e.s.d.'s involving l.s. planes.
Refinement. Refinement of *F*^2^ against ALL reflections. The weighted *R*-factor *wR* and goodness of fit *S* are based on *F*^2^, conventional *R*-factors *R* are based on *F*, with *F* set to zero for negative *F*^2^. The threshold expression of *F*^2^ > σ(*F*^2^) is used only for calculating *R*-factors(gt) *etc*. and is not relevant to the choice of reflections for refinement. *R*-factors based on *F*^2^ are statistically about twice as large as those based on *F*, and *R*-factors based on ALL data will be even larger.

### Fractional atomic coordinates and isotropic or equivalent isotropic displacement parameters (Å^2^)

**Table:**

	*x*	*y*	*z*	*U*_iso_*/*U*_eq_	
Mn1	0.0000	0.5000	0.5000	0.03697 (13)	
S1	0.00911 (6)	0.11697 (6)	0.14672 (6)	0.05653 (16)	
O1	0.24701 (13)	0.46530 (13)	0.41948 (12)	0.0385 (3)	
N1	0.48105 (16)	0.51194 (15)	0.24678 (14)	0.0354 (3)	
H1A	0.3779	0.5397	0.2737	0.043*	
O2	0.16157 (14)	0.30666 (15)	0.63303 (13)	0.0465 (3)	
C7	0.36297 (19)	0.35112 (18)	0.47429 (16)	0.0320 (3)	
C13	0.32723 (19)	0.25949 (19)	0.58901 (17)	0.0361 (4)	
C9	0.53201 (19)	0.31088 (19)	0.42564 (17)	0.0354 (4)	
C15	0.01407 (19)	0.2348 (2)	0.26679 (19)	0.0401 (4)	
C8	0.58045 (19)	0.3952 (2)	0.31424 (18)	0.0389 (4)	
H8A	0.6924	0.3648	0.2870	0.047*	
C4	0.5244 (2)	0.59880 (19)	0.13313 (17)	0.0358 (4)	
C3	0.6852 (2)	0.5520 (2)	0.06908 (19)	0.0451 (4)	
H3A	0.7678	0.4615	0.0990	0.054*	
C12	0.4481 (2)	0.1394 (2)	0.64754 (19)	0.0452 (4)	
H12A	0.4207	0.0812	0.7213	0.054*	
C5	0.4016 (2)	0.7315 (2)	0.08693 (19)	0.0441 (4)	
H5A	0.2935	0.7612	0.1290	0.053*	
C2	0.7210 (2)	0.6418 (2)	−0.0399 (2)	0.0537 (5)	
H2A	0.8288	0.6114	−0.0832	0.064*	
N2	0.01391 (19)	0.3211 (2)	0.35072 (18)	0.0554 (4)	
C10	0.6546 (2)	0.1866 (2)	0.4902 (2)	0.0475 (4)	
H10A	0.7647	0.1625	0.4585	0.057*	
C1	0.5992 (3)	0.7757 (2)	−0.0853 (2)	0.0551 (5)	
H1B	0.6248	0.8357	−0.1583	0.066*	
C11	0.6141 (2)	0.1025 (2)	0.5974 (2)	0.0514 (5)	
H11A	0.6960	0.0202	0.6380	0.062*	
C6	0.4396 (2)	0.8202 (2)	−0.0220 (2)	0.0529 (5)	
H6A	0.3570	0.9103	−0.0526	0.063*	
C14	0.1088 (3)	0.2163 (3)	0.7377 (3)	0.0723 (7)	
H14A	0.1511	0.2175	0.8256	0.108*	
H14B	0.1487	0.1096	0.7035	0.108*	
H14C	−0.0089	0.2609	0.7546	0.108*	

### Atomic displacement parameters (Å^2^)

**Table:**

	*U*^11^	*U*^22^	*U*^33^	*U*^12^	*U*^13^	*U*^23^
Mn1	0.02590 (19)	0.0477 (2)	0.0377 (2)	−0.01664 (16)	−0.00189 (14)	0.00535 (15)
S1	0.0503 (3)	0.0525 (3)	0.0652 (3)	−0.0210 (2)	0.0012 (2)	−0.0120 (2)
O1	0.0265 (6)	0.0450 (7)	0.0419 (6)	−0.0142 (5)	−0.0032 (5)	0.0135 (5)
N1	0.0272 (7)	0.0401 (8)	0.0386 (7)	−0.0153 (6)	0.0018 (6)	0.0017 (6)
O2	0.0352 (6)	0.0583 (8)	0.0466 (7)	−0.0229 (6)	−0.0025 (5)	0.0208 (6)
C7	0.0309 (8)	0.0344 (8)	0.0321 (8)	−0.0152 (7)	−0.0033 (6)	0.0011 (6)
C13	0.0345 (9)	0.0419 (9)	0.0347 (8)	−0.0188 (7)	−0.0038 (7)	0.0027 (7)
C9	0.0306 (8)	0.0381 (9)	0.0355 (8)	−0.0132 (7)	−0.0018 (6)	0.0021 (7)
C15	0.0272 (8)	0.0426 (10)	0.0477 (10)	−0.0136 (7)	0.0009 (7)	0.0048 (7)
C8	0.0263 (8)	0.0460 (10)	0.0418 (9)	−0.0139 (7)	0.0010 (7)	0.0012 (7)
C4	0.0367 (9)	0.0379 (9)	0.0359 (8)	−0.0199 (7)	0.0000 (7)	−0.0004 (7)
C3	0.0359 (9)	0.0495 (11)	0.0469 (10)	−0.0175 (8)	0.0016 (8)	0.0083 (8)
C12	0.0488 (11)	0.0445 (10)	0.0409 (9)	−0.0184 (8)	−0.0078 (8)	0.0120 (8)
C5	0.0382 (10)	0.0441 (10)	0.0468 (10)	−0.0154 (8)	−0.0008 (8)	0.0022 (8)
C2	0.0426 (10)	0.0636 (12)	0.0546 (11)	−0.0263 (10)	0.0068 (9)	0.0085 (9)
N2	0.0465 (9)	0.0663 (11)	0.0562 (10)	−0.0280 (8)	0.0035 (8)	−0.0098 (8)
C10	0.0306 (9)	0.0516 (11)	0.0512 (11)	−0.0089 (8)	−0.0038 (8)	0.0063 (8)
C1	0.0620 (13)	0.0575 (12)	0.0500 (11)	−0.0318 (11)	−0.0014 (9)	0.0142 (9)
C11	0.0422 (10)	0.0481 (11)	0.0531 (11)	−0.0073 (8)	−0.0115 (8)	0.0139 (9)
C6	0.0541 (12)	0.0450 (11)	0.0542 (11)	−0.0160 (9)	−0.0076 (9)	0.0124 (9)
C14	0.0556 (13)	0.0807 (16)	0.0782 (15)	−0.0333 (12)	0.0049 (11)	0.0363 (12)

### Geometric parameters (Å, °)

**Table:**

Mn1—O1	2.1455 (10)	C4—C5	1.379 (2)
Mn1—O1^i^	2.1455 (10)	C4—C3	1.384 (2)
Mn1—N2	2.1794 (16)	C3—C2	1.381 (2)
Mn1—N2^i^	2.1794 (16)	C3—H3A	0.9300
Mn1—O2	2.2525 (12)	C12—C11	1.406 (3)
Mn1—O2^i^	2.2525 (12)	C12—H12A	0.9300
S1—C15	1.6270 (19)	C5—C6	1.381 (3)
O1—C7	1.2942 (19)	C5—H5A	0.9300
N1—C8	1.297 (2)	C2—C1	1.378 (3)
N1—C4	1.420 (2)	C2—H2A	0.9300
N1—H1A	0.8600	C10—C11	1.351 (3)
O2—C13	1.3790 (19)	C10—H10A	0.9300
O2—C14	1.424 (2)	C1—C6	1.376 (3)
C7—C9	1.423 (2)	C1—H1B	0.9300
C7—C13	1.429 (2)	C11—H11A	0.9300
C13—C12	1.360 (2)	C6—H6A	0.9300
C9—C8	1.409 (2)	C14—H14A	0.9600
C9—C10	1.414 (2)	C14—H14B	0.9600
C15—N2	1.151 (2)	C14—H14C	0.9600
C8—H8A	0.9300		
			
O1—Mn1—O1^i^	180.0	C5—C4—C3	120.42 (16)
O1—Mn1—N2	90.41 (5)	C5—C4—N1	118.22 (14)
O1^i^—Mn1—N2	89.59 (5)	C3—C4—N1	121.36 (15)
O1—Mn1—N2^i^	89.59 (5)	C2—C3—C4	119.02 (17)
O1^i^—Mn1—N2^i^	90.41 (5)	C2—C3—H3A	120.5
N2—Mn1—N2^i^	180.00 (7)	C4—C3—H3A	120.5
O1—Mn1—O2	74.23 (4)	C13—C12—C11	120.51 (16)
O1^i^—Mn1—O2	105.77 (4)	C13—C12—H12A	119.7
N2—Mn1—O2	88.99 (6)	C11—C12—H12A	119.7
N2^i^—Mn1—O2	91.01 (6)	C4—C5—C6	119.83 (16)
O1—Mn1—O2^i^	105.77 (4)	C4—C5—H5A	120.1
O1^i^—Mn1—O2^i^	74.23 (4)	C6—C5—H5A	120.1
N2—Mn1—O2^i^	91.01 (6)	C1—C2—C3	120.86 (17)
N2^i^—Mn1—O2^i^	88.99 (6)	C1—C2—H2A	119.6
O2—Mn1—O2^i^	180.00 (5)	C3—C2—H2A	119.6
C7—O1—Mn1	116.71 (9)	C15—N2—Mn1	175.35 (17)
C8—N1—C4	126.92 (14)	C11—C10—C9	120.88 (16)
C8—N1—H1A	116.5	C11—C10—H10A	119.6
C4—N1—H1A	116.5	C9—C10—H10A	119.6
C13—O2—C14	119.03 (14)	C6—C1—C2	119.66 (18)
C13—O2—Mn1	114.02 (9)	C6—C1—H1B	120.2
C14—O2—Mn1	126.09 (12)	C2—C1—H1B	120.2
O1—C7—C9	122.31 (14)	C10—C11—C12	120.05 (17)
O1—C7—C13	121.33 (14)	C10—C11—H11A	120.0
C9—C7—C13	116.36 (14)	C12—C11—H11A	120.0
C12—C13—O2	124.73 (15)	C1—C6—C5	120.20 (18)
C12—C13—C7	121.84 (15)	C1—C6—H6A	119.9
O2—C13—C7	113.42 (14)	C5—C6—H6A	119.9
C8—C9—C10	118.78 (15)	O2—C14—H14A	109.5
C8—C9—C7	120.86 (15)	O2—C14—H14B	109.5
C10—C9—C7	120.35 (15)	H14A—C14—H14B	109.5
N2—C15—S1	178.24 (18)	O2—C14—H14C	109.5
N1—C8—C9	125.08 (14)	H14A—C14—H14C	109.5
N1—C8—H8A	117.5	H14B—C14—H14C	109.5
C9—C8—H8A	117.5		
			
N2—Mn1—O1—C7	−84.38 (11)	C13—C7—C9—C8	−179.21 (15)
N2^i^—Mn1—O1—C7	95.62 (11)	O1—C7—C9—C10	179.05 (15)
O2—Mn1—O1—C7	4.46 (10)	C13—C7—C9—C10	−0.2 (2)
O2^i^—Mn1—O1—C7	−175.54 (10)	C4—N1—C8—C9	−179.22 (15)
O1—Mn1—O2—C13	−4.72 (10)	C10—C9—C8—N1	−179.53 (16)
O1^i^—Mn1—O2—C13	175.28 (10)	C7—C9—C8—N1	−0.5 (3)
N2—Mn1—O2—C13	85.99 (11)	C8—N1—C4—C5	−172.09 (16)
N2^i^—Mn1—O2—C13	−94.01 (11)	C8—N1—C4—C3	8.3 (3)
O1—Mn1—O2—C14	−173.90 (17)	C5—C4—C3—C2	1.1 (3)
O1^i^—Mn1—O2—C14	6.10 (17)	N1—C4—C3—C2	−179.25 (16)
N2—Mn1—O2—C14	−83.19 (17)	O2—C13—C12—C11	−178.46 (16)
N2^i^—Mn1—O2—C14	96.81 (17)	C7—C13—C12—C11	0.8 (3)
Mn1—O1—C7—C9	176.96 (11)	C3—C4—C5—C6	−1.3 (3)
Mn1—O1—C7—C13	−3.79 (19)	N1—C4—C5—C6	179.06 (16)
C14—O2—C13—C12	−6.3 (3)	C4—C3—C2—C1	−0.3 (3)
Mn1—O2—C13—C12	−176.32 (13)	C8—C9—C10—C11	179.98 (17)
C14—O2—C13—C7	174.40 (17)	C7—C9—C10—C11	1.0 (3)
Mn1—O2—C13—C7	4.39 (17)	C3—C2—C1—C6	−0.5 (3)
O1—C7—C13—C12	−179.92 (15)	C9—C10—C11—C12	−0.9 (3)
C9—C7—C13—C12	−0.6 (2)	C13—C12—C11—C10	0.0 (3)
O1—C7—C13—O2	−0.6 (2)	C2—C1—C6—C5	0.3 (3)
C9—C7—C13—O2	178.68 (13)	C4—C5—C6—C1	0.6 (3)
O1—C7—C9—C8	0.1 (2)		

Symmetry codes: (i) −*x*, −*y*+1, −*z*+1.

### Hydrogen-bond geometry (Å, °)

**Table:**

*D*—H···*A*	*D*—H	H···*A*	*D*···*A*	*D*—H···*A*
N1—H1A···O1	0.86	1.97	2.6501 (16)	135
